# Subacute Thyroiditis Presenting as Idiopathic Intracranial Hypertension

**DOI:** 10.1155/2021/9203319

**Published:** 2021-12-20

**Authors:** Kanyanatt Boonyatarp, Kanoksri Samintharapanya, Thanawat Vongchaiudomchoke, Nuttaya Wachiraphansakul

**Affiliations:** ^1^Department of Internal Medicine, Lampang Hospital, Lampang, Thailand; ^2^Division of Neurology, Department of Internal Medicine, Lampang Hospital, Lampang, Thailand; ^3^Division of Nephrology, Department of Internal Medicine, Lampang Hospital, Lampang, Thailand; ^4^Division of Endocrinology and Metabolism, Department of Internal Medicine, Lampang Hospital, Lampang, Thailand

## Abstract

**Background:**

Several case reports have illustrated a rare neurological manifestation, idiopathic intracranial hypertension (IIH), in patients with thyrotoxicosis. However, none were diagnosed with thyroiditis. We report the case of a patient with subacute thyroiditis who presented with severe intractable headache due to IIH. *Case Presentation*. A 36-year-old woman visited Lampang Hospital in February 2021 complaining of neck pain and progressive severe intractable headache. Her vital signs and neurological examination were normal. Thyroid examination revealed a single 1 cm right thyroid nodule. A computed tomography (CT) scan of her brain illustrated diffuse brain edema. However, CT angiography and venography of the brain did not show abnormalities. The opening pressure of the cerebrospinal fluid was elevated (27 cmH_2_O). The free triiodothyronine level was 6.19 pg/mL, free thyroxine was 2.32 ng/dL, and thyroid-stimulating hormone was 0.0083 *μ*IU/mL. Anti-Tg was positive at a low titer, but anti-TPO was negative. TRAb was also negative. Methimazole and acetazolamide were prescribed and monitored. The symptoms resolved completely within 2 weeks of onset. Thyroid hormones had returned to normal by 8 weeks.

**Conclusion:**

This is the first case report of subacute thyroiditis presenting with IIH.

## 1. Introduction

Thyroiditis is the term used to describe thyroid gland inflammation that arises from any etiology. Therefore, there are variations in the clinical manifestations and course of thyroiditis. Its diagnosis is made by clinical symptoms of thyrotoxicosis with acute destruction of the thyroid gland, which leads to the release of the thyroid hormone into the systemic circulation. Thyroid gland tenderness is commonly found in cases of subacute thyroiditis and infectious thyroiditis but is rarely seen in Hashimoto's thyroiditis. However, no manifestations of idiopathic intracranial hypertension (IIH; also called pseudotumor cerebri) have been reported for the thyrotoxicosis stage. Here, we report the first case of subacute thyroiditis that presents as IIH.

## 2. Case Presentation

**Figure 1:**
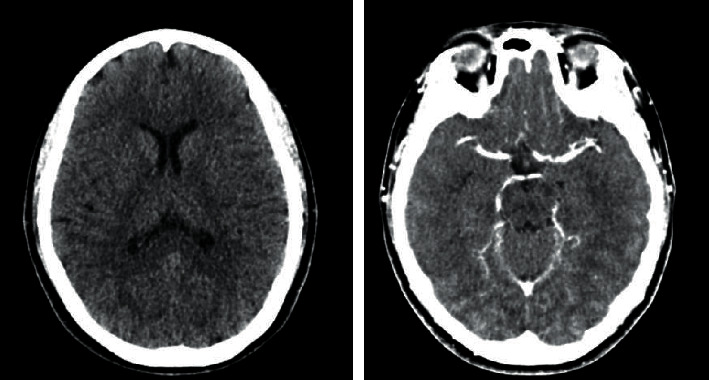
Brain computed tomography and venography revealed diffuse absence of cortical sulci without venous obstruction, consistent with diffuse cerebral edema.

**Figure 2:**
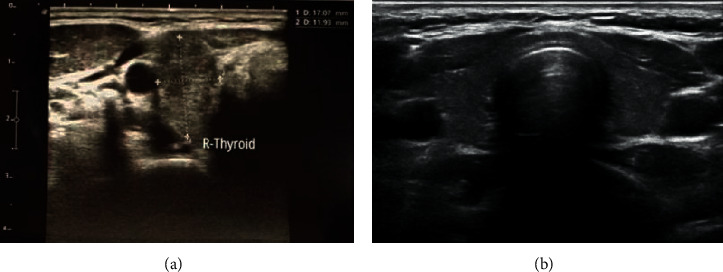
(a) A neck ultrasound showed a solitary 1 cm right thyroid nodule. (b) Follow-up neck ultrasound revealed no nodularity.

A 36-year-old woman visited Lampang Hospital in February 2021 complaining of a severe progressive intractable headache for 1 week. She also had dull aching shoulder and neck pain on the right side. These symptoms were not relieved with nonsteroidal anti-inflammatory drugs. She had no relevant medical history and was not taking any medications. On physical examination, her body temperature was 36.3°C. No clinical signs of hyperthyroidism were observed. Neurological examination was normal. There was no limitation of extraocular muscle movement, fundoscopic examination did not reveal papilledema, and the visual fields of both eyes were normal. Due to her painful neck, her thyroid function was evaluated on the first day of admission. The tests revealed primary hyperthyroidism: a free triiodothyronine level of 6.19 pg/mL (1.71–3.71 pg/mL), a free thyroxine level of 2.32 ng/dL (0.70–1.48 ng/dL), and a thyroid-stimulating hormone level of 0.0083 *μ*IU/mL (0.35–4.94 *μ*IU/mL) (Table 1). The results of a brain computed tomography (CT) scan illustrated a diffuse absence of cortical sulci, which was consistent with diffuse brain edema. Nevertheless, CT angiography and venography of her brain did not demonstrate abnormalities (Figure 1). The opening pressure of the cerebrospinal fluid (CSF) was elevated (27 cmH_2_O), and the CSF had normal levels of glucose and protein without cells. The erythrocyte sedimentation rate was high (51 mm/h). A workup was then conducted to determine the cause of thyrotoxicosis. A neck ultrasound showed a single 1 cm right thyroid nodule (Figure 2). However, she did not undergo thyroid scintigraphy due to recent administration of contrast media. While waiting for thyroid antibody results, methimazole (15 mg/day) and acetazolamide (750 mg/day) were prescribed. Potential etiologies of IIH were excluded by blood and CSF analyses (Table 1). Immunological investigation revealed a slightly elevated level of antithyroglobulin antibody (anti-Tg; 10.13 IU/ml). Antithyroid peroxidase antibody (anti-TPO) and antithyroid receptor antibody (TRAb) were both negative. With neck pain accompanied by thyrotoxicosis and a high erythrocyte sedimentation rate, subacute thyroiditis was the most likely diagnosis. Her headache gradually improved without the addition of steroid treatment, and it disappeared after 1 week. Acetazolamide was continued for 2 weeks, and she returned to the euthyroid state. Methimazole was tapered and discontinued after 6 weeks. Six months after the onset of thyrotoxicosis, her thyroid function was still normal, and her thyroid sonographic results did not reveal nodularity. A steady decline in the anti-Tg level was observed over the 6-month period, supporting the diagnosis of subacute thyroiditis (Table 1).

## 3. Discussion and Conclusions

Subacute thyroiditis, also known as de Quervain's thyroiditis, is believed to be caused by postviral inflammation that causes inflammation of the thyroid gland. The clinical course of subacute thyroiditis usually shows 3 consecutive phases that unfold over approximately 6 months. During the first weeks after the onset of symptoms, transient thyrotoxicosis is found as preformed thyroid hormones are released from the damaged gland. Within a few weeks, many patients enter a hypothyroid phase, which is later followed by euthyroid restoration [1]. Tenderness and goiter are common manifestations in most cases [2, 3]. A thyroid antibody workup should be performed to exclude Hashimoto's thyroiditis, which is one of the most common causes of thyroiditis [2]. Differential diagnosis between Graves' disease is also mandatory. In addition, TRAb levels must be measured as treatments for thyroiditis and Graves' disease differ [4].

The presentation in our case involved a painful thyroid enlargement without specific signs of Graves' disease. There was no exposure to contrast media before the first thyroid function workup. The TRAb results were negative. Although a low anti-Tg titer was found, subacute thyroiditis was the most likely diagnosis due to the clinical symptoms and the decrease in the thyroid titer over time.

Aside from tremor and mild agitation, neurological manifestations of thyroiditis are rarely seen. Steroid-responsive encephalopathy, known as Hashimoto encephalopathy, has been reported in individuals with cognitive or neuropsychiatric involvement accompanied by positive anti-TPO [5]. In our case, we demonstrated another neurological manifestation that was found simultaneously with thyroiditis: IIH. This combination of thyroiditis and IIH has not been previously reported.

IIH is not common, with an incidence rate of 0.9 cases per 100,000 population [6]. In nearly 90% of cases, no clearly identifiable etiology could be found. Thus, to make a diagnosis of IIH, other secondary causes of increased intracranial pressure must be investigated and ruled out. Brain magnetic resonance venography should be performed to exclude cerebral venous sinus thrombosis. The most well-known theory of IIH development is venous outflow obstruction or stenosis without thrombosis. Other theories, such as an increased CSF production rate or loss of cerebral autoregulation, have also been proposed [7–9].

There are only a few case reports of thyroid disorders and IIH. Two cases of Graves' disease were reported [10, 11]. Levothyroxine treatment of chronic hypothyroidism in children followed by the development of IIH was also reported [12]. Another 2 cases of levothyroxine treatment accompanied by the occurrence of IIH were reported in 2 patients with thyroid carcinoma after thyroidectomy [13].

Two mechanisms of thyroid hormone action and IIH have been postulated. First, thyroxine replacement might alter CSF flow due to normalization of excess water in body fluid composition, leading to correction of hyponatremia in the chronic hypothyroid state. Normalization of excess fluid in the CSF might alter the dynamics of the CSF and predispose patients to IIH [11]. Second, thyrotoxicosis leads to decreased cerebral vascular resistance, increased cerebral venous blood flow, and elevated venous blood pressure. As a consequence, failure of adequate drainage of the CSF from the subarachnoid space occurs, and IIH develops [11, 14].

In the present case, a postulated second mechanism is that transient overt thyrotoxicosis may have decreased cerebral vascular resistance and increased cerebral venous volume. Genetic susceptibility and structural abnormalities in venous drainage might also have played an important role, but they are still under investigation.

There are limitations to the investigations in this case. First, thyroid scintigraphy and fine-needle aspiration of thyroid tissue were not performed to definitively diagnose subacute thyroiditis. However, the diagnosis of subacute thyroiditis was the most likely cause of thyrotoxicosis in this patient due to the presence of a painful thyroid goiter in the first manifestation. Furthermore, there were no clinical clues of Graves' disease, and the TRAb level was normal. In the literature, a low positive titer (less than 100 IU/mL) of anti-Tg was also reported for subacute thyroiditis [15]. In the future, interval follow-ups of the clinical manifestations and the thyroid antibody levels should be carried out to determine the cause of thyroiditis. Second, to diagnose IIH, brain magnetic resonance venography was not performed in this case to assess cerebral venous drainage as it could detect venous stasis or early venous thrombosis. Brain CT venography was performed instead due to its widespread availability and acceptable sensitivity for detecting venous thrombosis (approximately 90%) [16, 17].

In conclusion, we demonstrated the first case of the thyrotoxicosis phase of thyroiditis with the unusual neurological manifestation of IIH. The mechanism and causal relationship between the 2 conditions are still unclear; however, these might be established by further investigations in the future.

## Figures and Tables

**Table 1:** Symptoms, laboratory results, and treatment by day of illness, hospital day, and follow-up day.

	Hospital day				Follow-up day				
	1	2	3	4	11	24	38	68	218
Day of illness	D8	D9	D10	D11	D18	D31	D45	D85	D225
Neck pain	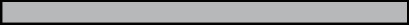								
Headache	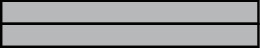								
Vomiting									
Body temperature (°C)	36.3	36.7	37.2	36.9	36.9	36.2	37.0	36.5	36.5
Hemoglobin (g/dL)	10.2	9.9	11.3		12.7	12.0	12.9	12.7	
White blood cell count (/cu.mm)	5400	7400	6900		5500	5500	4700	4800	
Neutrophil (%)	76	74	67		61	71	60	55	
Lymphocyte (%)	17	18	22		27	22	32	34	
Platelet count (×10^3^/cu.mm)	287,000	269,000	346,000		399,000	317,000	324,000	273,000	
Free T3 (pg/mL) (1.71–3.71)	6.19				7.07	2.86	2.15	2.07	2.23
Free T4 (ng/dL) (0.70–1.48)	2.32				2.20	1.18	0.66	0.87	0.98
TSH (*μ*IU/mL) (0.350–4.940)	0.0083				0.0040	0.0047	2.18	4.39	3.16
Anti-Tg (IU/mL)	10.13								5.28
Anti-TPO (IU/mL)	<3.00								0.25
TRAb (IU/L)	<0.8								
ESR (mm/hr)		51							6
hs-CRP (mg/L)		41.4							
BUN (mg/dL)	15	15	13		120				
Creatinine (mg/dL)	0.57	0.49	0.70		0.54	0.54			
Sodium (mmol/L)	143	142	138		139	140			
Potassium (mmol/L)	4.1	3.8	3.8		3.8	3.7			
Chloride (mmol/L)	110	111	111		110	107			
Bicarbonate (mmol/L)	28	24	18		16	25			
CSF opening pressure (cmH_2_O)	27		18						
Glucose CSF	52								
Protein CSF	23.9								
Color	Colorless								
RBC	No RBC								
Cell count	No cell								
Therapy									
Methimazole	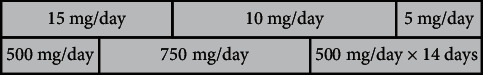								
Acetazolamide									

Anti-Tg: antithyroglobulin antibody; anti-TPO: antithyroid peroxidase antibody; BUN: blood urea nitrogen; CSF: cerebrospinal fluid; ESR: erythrocyte sedimentation rate; hs-CRP: high-sensitivity C-reactive protein; T3: triiodothyronine; T4: thyroxine; TRAb: antithyroid receptor antibody.

## Data Availability

All of the patient's data including outpatient department visit and inpatient admission data are available at Lampang Hospital, Lampang, Thailand.
